# Identification of Malignancy-Associated Changes in Histologically Normal Tumor-Adjacent Epithelium of Patients with HPV-Positive Oropharyngeal Cancer

**DOI:** 10.1155/2018/1607814

**Published:** 2018-03-11

**Authors:** James Jabalee, Anita Carraro, Tony Ng, Eitan Prisman, Cathie Garnis, Martial Guillaud

**Affiliations:** ^1^British Columbia Cancer Research Centre, Department of Integrative Oncology, Vancouver, BC, Canada; ^2^Department of Pathology, Vancouver General Hospital and University of British Columbia, Vancouver, BC, Canada; ^3^Vancouver General Hospital, Division of Otolaryngology-Head and Neck Surgery, Vancouver, BC, Canada; ^4^University of British Columbia, Vancouver, BC, Canada

## Abstract

The incidence of HPV-positive oropharyngeal cancer (HPV+ OPC) is increasing, thus presenting new challenges for disease detection and management. Noninvasive methods involving brush biopsies of diseased tissues were recently reported as insufficient for tumor detection in HPV+ OPC patients, likely due to differences between the site of tumor initiation at the base of involuted crypts and the site of brush biopsy at the crypt surface. We hypothesized that histologically normal surface epithelial cells in the oropharynx contain changes in nuclear morphology that arise due to tumor proximity. We analyzed the nuclear phenotype of matched tumor, tumor-adjacent normal, and contralateral normal tissues from biopsies of nine HPV+ OPC patients. Measurements of 89 nuclear features were used to train a random forest-based classifier to discriminate between normal and tumor nuclei. We then extracted voting scores from the trained classifier, which classify nuclei on a continuous scale from zero (“normal-like”) to one (“tumor-like”). In each case, the average score of the adjacent normal nuclei was intermediate between the tumor and contralateral normal nuclei. These results provide evidence for the existence of phenotypic changes in histologically normal, tumor-adjacent surface epithelial cells, which could be used as brush biopsy-based biomarkers for HPV+ OPC detection.

## 1. Introduction

Oropharyngeal cancers (OPCs), which include malignancies of the tonsils, posterior pharyngeal wall, soft palate, and tongue base, have undergone a dramatic epidemiological shift. While the rate of tobacco and alcohol-related head and neck cancers is declining in North America, the incidence of HPV-positive OPC (HPV+ OPC) has been steadily rising since the early 1980s and is especially prevalent among young individuals (<60 years of age) [1]. These observations highlight the need for improved means of detecting and managing the disease.

The current standard of care, chemoradiation therapy (CRT), is associated with severe negative side effects, both acute and chronic [2–4]. Given the relatively young age and high survival rate of this cohort, patients often must live with significant morbidities for decades [5]. To avoid the drawbacks of CRT, transoral robotic surgery (TORS), in which the tumor is resected through the mouth, is currently being investigated as an alternative treatment option [6]. However, to facilitate TORS and circumvent the need for concurrent CRT and its associated toxicities, tumors must be diagnosed at an early stage (T3 or less) [6]. Thus, a method of screening for the disease in high-risk populations would increase the number of TORS-eligible patients, resulting in reduced morbidity and improved patient quality of life.

Previous work attempted to create a noninvasive screening method for HPV+ OPC by sampling the oropharyngeal epithelium via brush biopsy and testing for the presence of HPV16 DNA via PCR [7]. Unfortunately, viral DNA was present only in a subset of HPV+ OPC patients in which the tumor tissue was directly accessible, and not in those where disease did not present at the oropharyngeal surface [7]. The most likely explanation is that oropharyngeal tumors often initiate at the base of deep invaginations, or crypts, whereas brushing samples only the normal surface epithelial cells, which do not exhibit HPV infection [8]. Identifying biomarkers present in the superficial epithelium adjacent to a tumor is an essential step in the development of noninvasive, brush biopsy-based screening methods for detecting HPV+ OPC.

Malignancy-associated changes (MACs) are subtle morphological changes that occur in histologically normal cells due to their proximity to a tumor and may include changes in the size, shape, and chromatin structure of the nucleus [9]. MACs have been shown to be reproducibly measured via image cytometry for numerous cancer types, including lung [10], breast [11], colon [12], cervical [13, 14], and oral [15–17] cancer, thus making them potentially useful diagnostic biomarkers. Here we use an in-house imaging system to measure >100 nuclear features for each cell nucleus of epithelial tissue samples derived from HPV+ OPC patients to determine their utility as diagnostic biomarkers.

## 2. Materials and Methods

### 2.1. Sample Source

Nine tumors from OPC patients were collected via head and neck surgeries performed at Vancouver General Hospital as part of a randomized control trial comparing TORS to radiation to treat early-stage OPC. The use of this tissue was approved by the University of British Columbia Ethics Committee (ID#: H15-01121). Informed consent was obtained from each patient prior to surgery. Eligible patients had tumors < 4 cm in diameter (stage T1/T2) and lymph node metastases of <3 cm on either side of the neck (stage N0/N1/N2). A biopsy of the tumor, including adjacent histologically normal surface epithelium, and a biopsy of contralateral normal tissue were collected from each patient. Patient demographics are included in Table S1.

### 2.2. Sample Preparation

**Figure 1:**
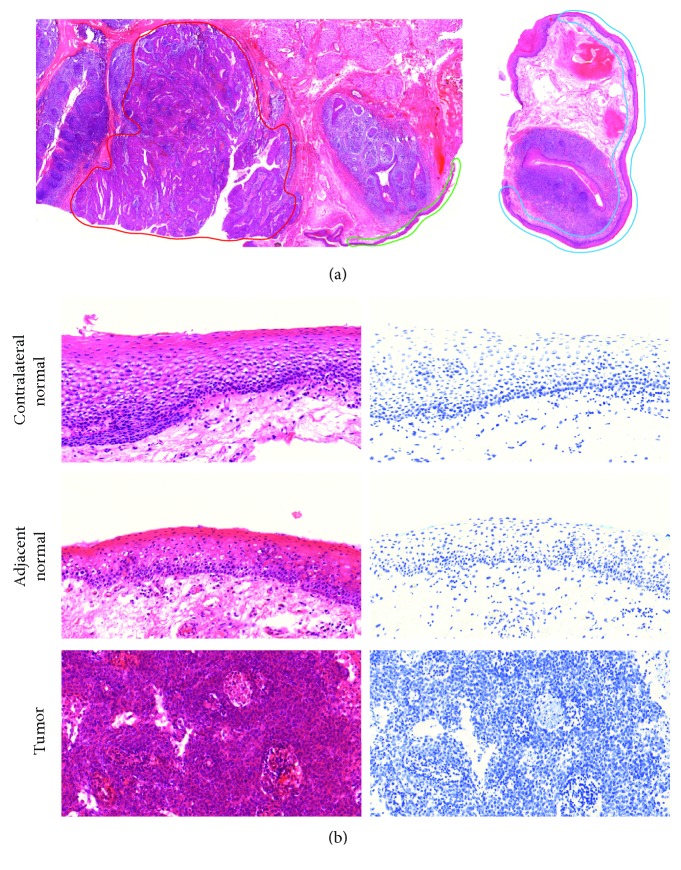
Regions of interest. (a) Tumor (left) and contralateral normal (right) biopsies were collected at the time of surgery. Three regions of interest corresponding to the tumor (red), adjacent normal epithelium (green), and contralateral normal epithelium (blue) were outlined by the study pathologist and used for analysis. (b) Close-up of each region of interest stained with H&E (left) or Feulgen-thionin (right).

Nondiagnostic tissues were fixed in molecular fixative by the study pathologist, Dr. T. Ng, and paraffin embedded. Four-micrometer-thick sections were cut, and a subset was stained with hematoxylin and eosin (H&E). These slides were used by the pathologist to identify three areas of interest: (1) tumor, (2) adjacent normal epithelium (an), and (3) contralateral normal epithelium (cln) (Figure 1). Sections immediately neighboring the H&E-stained slides were stained with Feulgen-thionin [18], which is stoichiometric for DNA, and areas of interest were imaged as described below.

### 2.3. Image Cytometry

A semiautomated quantitative imaging system was used [19, 20]. Regions of interest identified in H&E-stained slides were manually delineated in adjacent Feulgen-thionin-stained sections by an experienced technician. The imaging system then automatically located and focused individual objects, imaged them, and classified them as intact, in-focus, or “junk” nuclei using a random forest-based algorithm as previously described [17]. Nuclei spanning the full width of the epithelium were included in the analysis. The result of this process was then confirmed by an experienced technician. An illumination wavelength of 600 ± 5 nm was used, corresponding to the absorption peak of the Feulgen-thionin stain. An effective pixel sampling space in the plane of the sample of 0.34 *μ*m^2^ and an effective pixel sampling area of 0.116 *μ*m^2^ were used. Imaging system characteristics were in conformity with the European Society of Analytical Cellular Pathology [21]. For each nucleus, the software calculated >100 features related to size, shape, DNA quantity, and chromatin distribution as previously described [22]. Features found to be highly sensitive to the variability in staining intensity as assessed by our own experience were removed prior to the final analysis.

### 2.4. Nuclear Classification

A random forest learning algorithm was used to classify in-focus and intact nuclei as belonging to either contralateral normal or tumor tissue. This tree-based classification algorithm has the advantage of being robust against overfitting [23] and has been previously shown to perform well on a similar data set [17]. Nuclei from tumor and contralateral normal, but not adjacent normal, epithelial cells were used to train the classifier. Nuclei were randomly split into training (80%) and test (20%) sets. The number of features used by the classifier was tuned using 10-fold cross-validation (Table 1). Five hundred random trees were generated, and subsampling was done without replacement. Once the relevant parameters had been tuned, model performance was assessed on the test set using a receiver operating characteristic (ROC) curve [24].

Although all nuclei derived from normal tissue can be considered normal, not all nuclei derived from tumor tissue can be considered abnormal. Indeed, tumors are composed of a collection of cell types, including stromal cells, lymphocytes, fibroblasts, and others in addition to cancer cells, and the proportions of these cell types vary by tumor. Thus, any nucleus from tumor tissue that is classified as abnormal is considered more likely to have been derived from abnormal tissue than normal tissue. We selected only those epithelial nuclei derived from abnormal regions of tumor tissue for further analysis.

### 2.5. Large-Scale DNA Organization (LDO) Score

We extracted the voting score generated by the random forest classifier, which we term the large-scale DNA organization (LDO) score. The LDO score refers to the proportion of trees that classify a nucleus as “normal-like” and ranges from 0 (all trees vote “normal-like”) to 1 (no trees vote “normal-like”). Intermediate scores represent a phenotype intermediate between “normal-like” and “tumor-like.”

### 2.6. Statistical Analysis

Statistical analyses were performed using R statistical software (version 3.2.5). The random forest algorithm was implemented, parameters were tuned, and performance was evaluated using the CARET package [25], and ROC curves were generated using the pROC package [26]. For box plots, the center line of the box represents the median, and the box limits represent the 25th and 75th percentiles. The upper and lower whiskers extend to the 5th and 95th percentiles.

## 3. Results

A total of 4830 tumor and 3352 contralateral normal nuclei were used to train the random forest classifier. When applied to the test set, the model showed an area under the ROC curve of 0.90. A total of 84.7% of the 1207 tumor and 80.8% of the 837 contralateral normal test set nuclei were correctly classified (Table 2).

**Figure 2:**
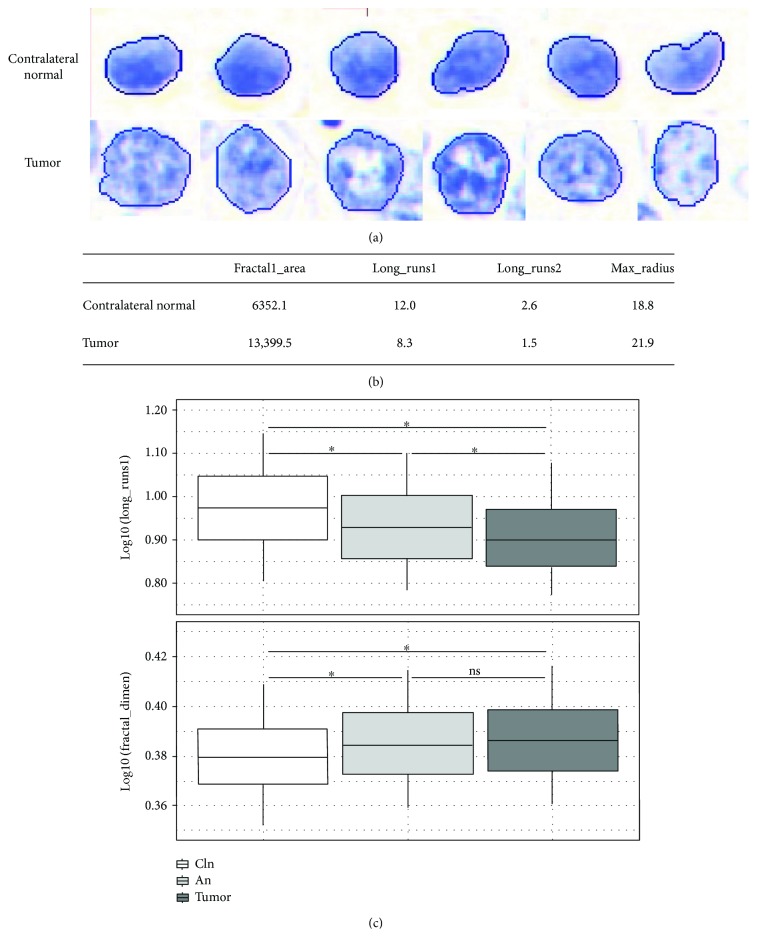
Examples of nuclear morphology of contralateral normal and tumor cells and the distribution of select features. (a) Images of Feulgen-thionin-stained nuclei. (b) Mean of select features for the nuclei pictured in (a). (c) Distribution of select features in contralateral normal (cln), adjacent normal (an), and tumor nuclei. Significance tested using ANOVA of log-10-transformed values with Tukey's post hoc honestly significant difference test. ^∗^ *p* < 0.001. ns: not significant.

Examples of contralateral normal and tumor nuclei and values of select features are given in Figures 2(a) and 2(b). Of the top ten most important features of our model (based on the mean decrease in accuracy), eight represent chromatin texture features, while the remainder represent morphological and photometric features (the meaning of each feature is given in Table S2; for additional details, see Doudkine et al. [22]). All ten features are statistically significantly different between contralateral normal and tumor nuclei (two-tailed *t*-test of log-10-transformed values with Benjamini-Hochberg correction, *p* < 0.001). If cells of the adjacent normal epithelium do not contain MACs, we would expect that there would be no significant difference between contralateral normal and adjacent normal nuclei for any feature. However, of the top 10 most important model features, we find that four (fractal1_area, long_runs2, run135_percent, and long_runs1) show an intermediate phenotype between that of the contralateral normal and tumor (i.e., a statistically significant difference between adjacent normal and contralateral normal, and between adjacent normal and tumor), while three (max_radius, long135_runs, and fractal_dimen) are significantly different from the contralateral normal but not the tumor (ANOVA of log-10-transformed values with Tukey's post hoc honestly significant difference test, *p* < 0.001; Figure 2(c)).

**Figure 3:**
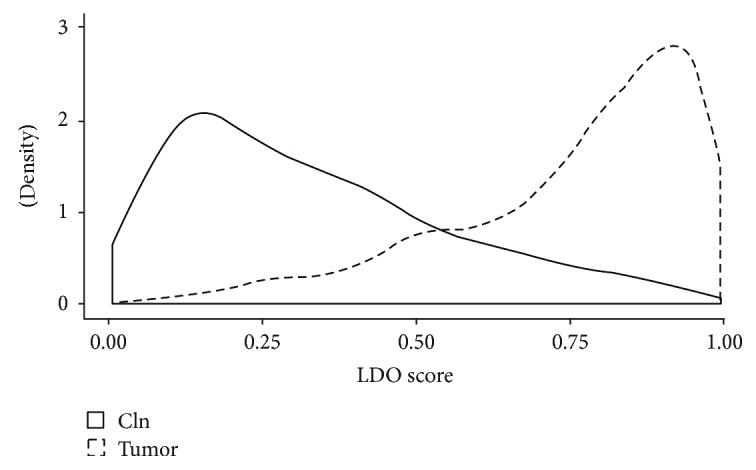
Distribution of LDO scores of nuclei derived from contralateral normal (cln) and tumor tissue of the test set.

**Figure 4:**
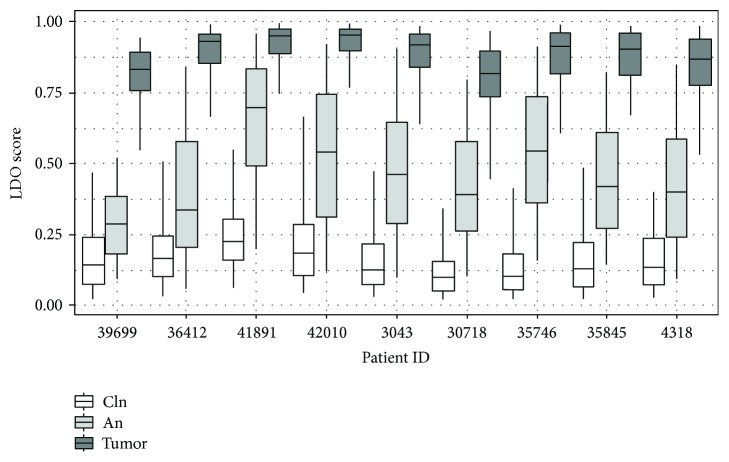
Distribution of the LDO scores of nuclei derived from contralateral normal (cln), adjacent normal (an), and tumor tissue grouped by patient. In each case, the mean LDO of the adjacent normal epithelium is intermediate between that of the contralateral normal and the tumor.

Large-scale DNA organization (LDO) scores are a measure of how “normal-like” or “tumor-like” a specific nucleus is within the context of our model. The distribution of LDO scores of tumor and contralateral normal samples of the test set is shown in Figure 3. Contralateral normal samples are skewed to the right while tumor samples are skewed to the left, indicating that nuclei from tumor-derived epithelial cells are more likely to be abnormal. To further demonstrate that adjacent normal epithelial cells contain MACs, we used our model to calculate the LDO scores for the full data set consisting of 4189 contralateral normal, 5192 adjacent normal, and 6037 tumor nuclei from nine patients. If adjacent normal nuclei contain MACs, then the distribution of LDO scores should be intermediate between that of the contralateral normal and tumor. For each patient, the mean of the distribution of LDO scores from contralateral normal epithelium is close to zero (0.16 ± 0.15) and that of tumor is close to one (0.88 ± 0.13), as expected, whereas adjacent normal nuclei display an intermediate phenotype (0.49 ± 0.25; Figure 4). Furthermore, the distribution of LDO scores of adjacent normal nuclei is statistically significantly different from both the contralateral normal and tumor for each of the nine patients (ANOVA with Tukey's post hoc honestly significant difference test, *p* < 0.001). These results strongly suggest the presence of MACs in histologically normal tumor-adjacent epithelial cell nuclei.

## 4. Discussion

The rising incidence of HPV+ OPC presents new challenges for the detection and management of oropharyngeal malignancies. Given the relatively young age and good health of this cohort, much attention is being given to methods that deescalate the intensity of therapy without sacrificing its effectiveness, thus reducing morbidity and improving long-term patient quality of life [27]. TORS is a promising means for achieving this goal as it provides an alternative to standard CRT for individuals with early-stage tumors. TORS has similar disease outcomes when compared to CRT [28–30] but with improved functional outcomes [28–35]. Importantly, TORS has been found to result in significantly lower rates of dysphagia requiring either a gastrostomy tube or tracheotomy [28, 34], which are among the most common and challenging complications associated with CRT [2, 3, 34]. Strikingly, Moore et al. report preservation of swallowing function in >90% of TORS patients [28], whereas the rate of dysphagia requiring a gastrostomy tube following CRT approaches 45% [3, 36]. Patients treated with TORS also tend to score higher on factors related to quality of life, including eating, speech, and social, than did those treated with adjuvant radiation therapy or chemoradiation therapy [31, 32, 35]. Unfortunately, the use of TORS is limited to patients with early-stage tumors exhibiting limited nodal involvement [6]. A method of screening high-risk individuals would increase the number of TORS-eligible patients, thus avoiding the need for CRT and improving patient quality of life.

We sought to determine if MACs exist in cells of the histologically normal oropharyngeal surface epithelium that exists adjacent to tumor tissues. Previous reports describe MACs as being robustly identified in patients with early-stage tumors at other organ sites, making them ideal candidates for early tumor detection [11, 15, 37–41]. Furthermore, surface epithelial cells are easily accessible via brush biopsy, which is an attractive sampling approach for the oropharynx as it is cheap, rapid, safe, painless, and noninvasive. Although previous studies have reported that brush biopsies are problematic for general OPC screening because they cannot directly sample tumors arising in tonsillar crypts [7], brush biopsies of clearly visible and accessible OPC lesions have been reported to be effective for HPV detection [7, 42]. Further, brush biopsies in the adjacent oral cavity, where tissues are less convoluted, have been reported to have high sensitivity and specificity for the detection of DNA aneuploidy, a nuclear morphological marker of neoplastic cell transformation [43]. Thus, the identification of MACs in surface epithelium represents the initial step towards the development of a brush biopsy-based screening method for early-stage oropharyngeal tumors in high-risk populations. Additional research is required to determine if the differences in nuclear phenotype observed in this study can be similarly identified in epithelial cells collected via brush biopsy.

Image cytometry shows great clinical promise and has been adopted for the screening of cervical cancer with great success [44]. We used image cytometry to measure nuclear features of contralateral normal, adjacent normal, and tumor epithelial cell nuclei to identify phenotypic differences among them. Of the ten most important nuclear features identified by our model (based on the mean decrease in accuracy), eight were associated with chromatin texture (Table S1). Changes in chromatin are known to accompany malignant transformation and have recently been shown to influence gene expression [45] and genome stability [46], suggesting an active role for chromatin reorganization in tumorigenesis. Adjacent normal epithelial cells show values intermediate between the contralateral normal and tumor for a number of these features, including fractal1_area (which measures the intensity contrast between condensed and uncondensed chromatin, thus revealing the organization of heterochromatin and euchromatin) and long_runs1 (which measures the random distribution of chromatin within the nucleus). Notably, since the contralateral normal epithelium used in our study was obtained from patients with OPC tumors, it is possible that these cells also contain MACs, but to a lesser degree than adjacent normal cells. MACs have been described in cells contralateral to the tumor of lung cancer patients [10], demonstrating that long-range effects are possible. Thus, analysis of cells from epithelia of individuals without cancer is required to overcome this issue.

Additional evidence for the existence of MACs in adjacent normal epithelial cells comes from our observation that the average LDO score of the adjacent normal epithelium is intermediate between that of the contralateral normal and tumor for each patient examined. Similarly, hyperplastic and mild/moderate dysplastic cells of the oral cavity also display an average LDO score intermediate between that of normal and tumor tissues [17]. Increased LDO is associated with molecular alterations such as loss of heterozygosity [47] and correlates with an increased risk of cancer progression [17, 47]. Interestingly, the standard deviation of the distribution of LDO scores for adjacent normal nuclei is consistently higher than that of either contralateral normal or tumor nuclei, suggesting greater heterogeneity in nuclear phenotype among adjacent normal epithelial cells.

Even with a small patient cohort, we provide strong evidence that the overall chromatin organization of adjacent normal cells differs from that of the contralateral normal and tumor. Since adjacent normal and contralateral normal epithelia are histologically identical and derive from the same anatomical structure (i.e., surface oropharyngeal epithelium), it is reasonable to conclude that the observed differences are due to the differing proximity of each epithelium to the tumor, a finding that conforms to the definition of MACs.

## 5. Conclusions

Malignancy-associated changes are reliably detected in histologically normal (normal-appearing) oropharyngeal epithelial cells located adjacent to a tumor and could be used as a noninvasive means of detecting early-stage oropharyngeal tumors.

## Figures and Tables

**Table 1:** Parameters of the nuclear classification model.

Initial group	Classification groups	# of trees	# of features per tree	Sample size	Sampling
Intact, in-focus nuclei identified by technician	Contralateral normal, tumor	500	35	Contralateral normal: 5572 Tumor: 6653	Without replacement

**Table 2:** Performance of the nuclear classification model.

		Predicted		Correct classification rate (%)
		Contralateral normal	Tumor	
A: Training set				
Actual	Contralateral normal	**2519**	883	75.5
	Tumor	661	**4169**	86.3
B: Test set				
Actual	Contralateral normal	**647**	154	80.8
	Tumor	190	**1053**	84.7

Correctly classified nuclei represented in bold.

## Supplementary Materials

Supplementary Materials Supplementary Table 1: it provides an overview of patient demographics for all individuals included in this study. Supplementary Table 2: it provides a description of the most important nuclear features as defined by our model.
