# Supplementary material for: Identification of Malignancy-Associated Changes in Histologically Normal Tumor-Adjacent Epithelium of Patients with HPV-Positive Oropharyngeal Cancer
Source: Anal Cell Pathol (Amst). 2018 Mar 11;2018:1607814. doi: 10.1155/2018/1607814 (PMC5866869; doi:10.1155/2018/1607814)
Supplement: Supplementary Materials — Supplementary Table 1: it provides an overview of patient demographics for all individuals included in this study. Supplementary Table 2: it provides a description of the most important nuclear features as defined by our model. [file 1607814.f1.pdf]

SUPPLEMENTARY INFORMATION

Table S1 – Patient demographics.

Patient ID	Age	Sex	Site	Ethnicity
**39699**	52	M	Tonsil	Caucasian
**36412**	67	M	Tonsil	Caucasian
**41891**	55	M	Tongue base	Caucasian
**42010**	54	M	Tonsil	Caucasian
**3043**	60	M	Tongue base	Caucasian
**4318**	64	M	Tonsil	Caucasian
**30718**	69	M	Tonsil	Caucasian
**35746**	61	M	Tongue base	Caucasian
**35845**	46	M	Tonsil	Caucasian

Table S2 – Interpretation of important model features.

Feature	Type (subtype)	Interpretation
fractal1_area	Texture (fractal)	Measurement of the area of a three-dimensional surface, created by the nuclear optical density function.
long_runs2	Texture (run length)	Standard deviation of long_runs, which gives large values for objects in which consecutive, collinear pixels having the same grey level value dominate
correlation	Texture (Markovian)	A large value indicates an object with large connected subcomponents of constant grey level and with large grey level differences between adjacent components.
long135_runs	Texture (run length)	Gives a large value for objects in which consecutive, collinear pixels having the same grey level value oriented at 135 degrees dominate.
run135_percent	Texture (run length)	Ratio of the total number of possible runs to the object's area, having its lowest value for pictures with the most linear structure.

fractal2_area	Texture (fractal)	Similar to fractal1_area, but based on an image in which four pixels are averaged into a single pixel, thereby representing a change of scale of fractal1_area.
long_runs1	Texture (run length)	Mean of long_runs, which gives large values for objects in which consecutive, collinear pixels having the same grey level value dominate.
max_radius	Morphological	Maximum value of the length of the measurement of the smoothness of image intensity – large for nuclei with slight and spatially smooth grey level variations.
OD_kurtosis	Photometric	The normalized fourth moment of the optical density function of the object.
fractal_dimen	Texture (fractal)	Difference between log(fractal1_area) and log(fractal2_area), divided by log(2). Varies from 2 to 3 and gives a measure of the “fractal behavior” of the image, associated with a rate at which measured surface area increases at finer and finer scales.
